# Two Faces of NFAT Transcription Factors in Lymphocytes*—A Personal Account*

**DOI:** 10.3390/biom16060757

**Published:** 2026-05-22

**Authors:** Edgar Serfling

**Affiliations:** 1 Department of Molecular Pathology, Institute of Pathology, University of Würzburg, Josef-Schneider-Str. 2, 97080 Würzburg, Germany; serfling.e@mail.uni-wuerzburg.de; 2 Comprehensive Cancer Center Mainfranken, University of Würzburg, Josef Schneider-Str. 6, 97080 Würzburg, Germany

**Keywords:** NFAT, transcription factors, T cells

## Abstract

The two NFAT transcription factors NFATc1 and NFATc2 are the most prominent Ca^++^-dependent TFs in the nuclei of activated peripheral lymphocytes. They control the activity of thousands of genes during immune responses. Although their structure and function show numerous things in common, their expression and activity differ markedly in most types of lymphocytes. Over the last 40 years, the work of our laboratory revealed a strong inducible transcription of the *Nfatc1* gene upon lymphocyte (co-)activation, compared to the ‘tonic’ transcription of *Nfatc2*. This leads to the inducible expression of a short NFATc1 isoform that we designated as NFATc1/αA, which differs from longer NFATc1 proteins and NFATc2 by an individual N-terminal ‘α’ peptide and the absence of a C-terminal peptide of approximately 250 amino acid residues. While comprehensive experimental studies led to the conclusion that NFATc2 supports (i) apoptosis, (ii) the induction of anergy, and (iii) the ‘exhaustion’ of peripheral T cells, opposite conclusions can be derived from our studies of NFATc1/αA. This view on the ‘two faces’ of NFAT transcription factors will be presented in this review and discussed in the role of NFATs in cancerogenesis.

## 1. Introduction: A Brief History of Early NFAT Research

NFAT (‘Nuclear Factor of Activated T Cell’) transcription factors (TFs) were first described as nuclear proteins that bind upon activation of human and murine T cells to the interleukin 2 (*Il2*) promoter/enhancer by Jerry Crabtree and our laboratory in 1988/89 [1,2]. Soon after, both laboratories described this activity as cyclosporin A (CsA)-sensitive (i.e., CsA treatment of human Jurkat or murine EL-4 leukemia T cells specifically inhibited the binding of this factor to the two ‘purine boxes’ of the *Il2* promoter*) [3,4].

The name NF-AT (‘Nuclear Factor of Activated T Cells’) was coined by G. Crabtree, in analogy to NF-κB described before by Sen and Baltimore [5]. Since at that time we knew that NFATs are also expressed in B cells [6], we preferred to call this activity “Pu-box factor” [2,4], but soon followed Crabtree’s recommendation.

The circular oligopeptide CsA was isolated from the fungus Tolypocladium by the Swiss scientists Borel and Stähelin more than a decade before and revolutionized transplant medicine [7,8]. By the inhibition of immune reactions, CsA prevented the rejection of transplanted organs, but at that time “…no one knew how CsA works at the molecular level” [9]. The specific inhibitory effect of low doses of CsA on the binding of NFAT factors to the “purine boxes” of the *Il2* promoter suggested that CsA acts by suppressing the activity of NFATs. This assumption was corroborated by the binding of CsA to calcineurin (CN), a Ca^++^-dependent protein phosphatase [10]. In lymphoid (and other) cells, both CsA and its functional ‘sibling’ FK506 (Tacrolimed) bind to low molecular weight proteins, the immunophilins, and these complexes bind, along with calmoldulin, to the large A subunit of CN and inhibit CN’s enzymatic activity. Overexpression of CN in T cells leads to a strong increase in *Il2* promoter activity, suggesting that NFAT factors are prominent targets of CN in lymphocytes [11,12].

**Figure 1:**
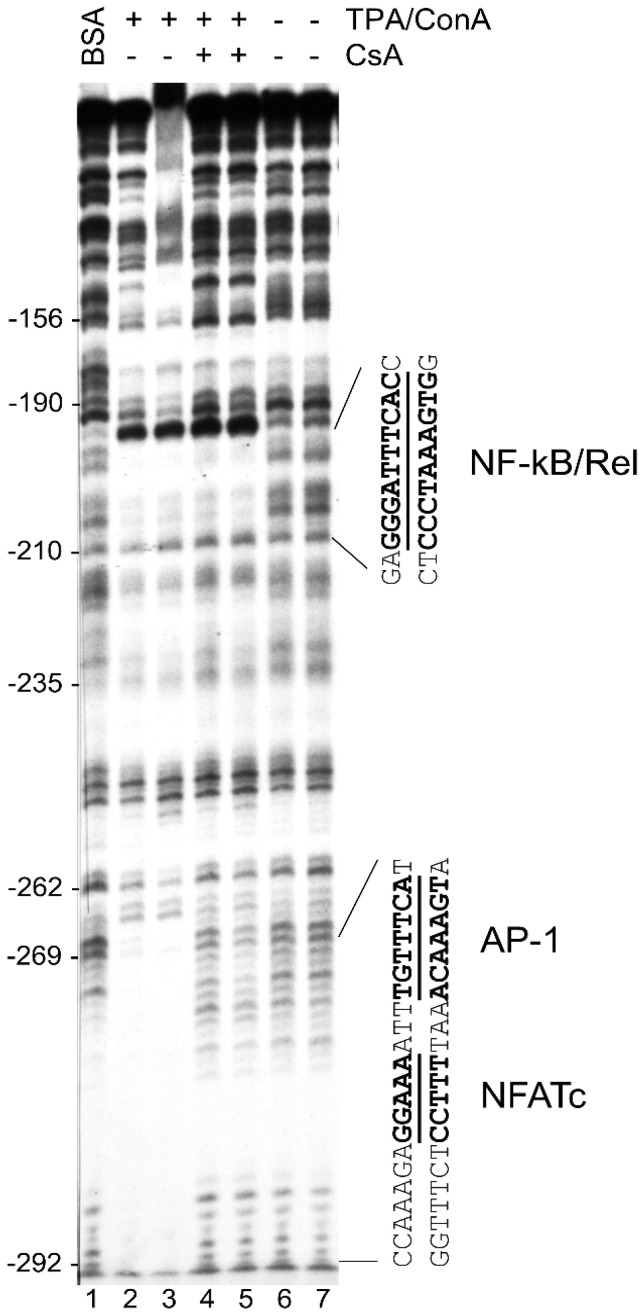
Identification of CsA-sensitive binding of NFATs to the murine *Il2* promoter. Autoradiography of DNase I footprint assays using nuclear proteins from EL-4 leukemia cells that were stimulated by TPA + ionomycin for 24 h (+) without or with CsA (+/CsA), or were left unstimulated (−). The extracts were incubated for 60 min with ^32^P-labeled probes of murine *Il2* promoter spanning the nucleotides from position −7 to −293, followed by a short DNase I treatment, stop for incubation, phenol treatment, ethanol precipitation and electrophoresis on a 6% polyacrylamide-urea gel (for details see refs. [2,4]). Note that the AP-1 and NF-kB/Rel sites differ slightly from ‘canonical’ binding sites which contribute to the T cell specificity of *Il2* promoter activity [13].

The CsA-sensitive binding of NFATs to the human and murine *Il2* promoter was demonstrated in DNase I footprint protection assays and EMSAs using nuclear proteins from activated human Jurkat and murine EL-4 cells. NFAT binding was detected upon treatment of T cells by TPA (PMA) and ionomycin for one day, whereas simultaneous treatment by low doses of CsA (less than 100 ng CsA per ml culture medium) blocked any NFAT binding. In leukemic T cells, the binding of other factors, such as of NF-kB factors, to the *Il2* promoter remained unaffected by CsA (Figure 1).

**Figure 2:**
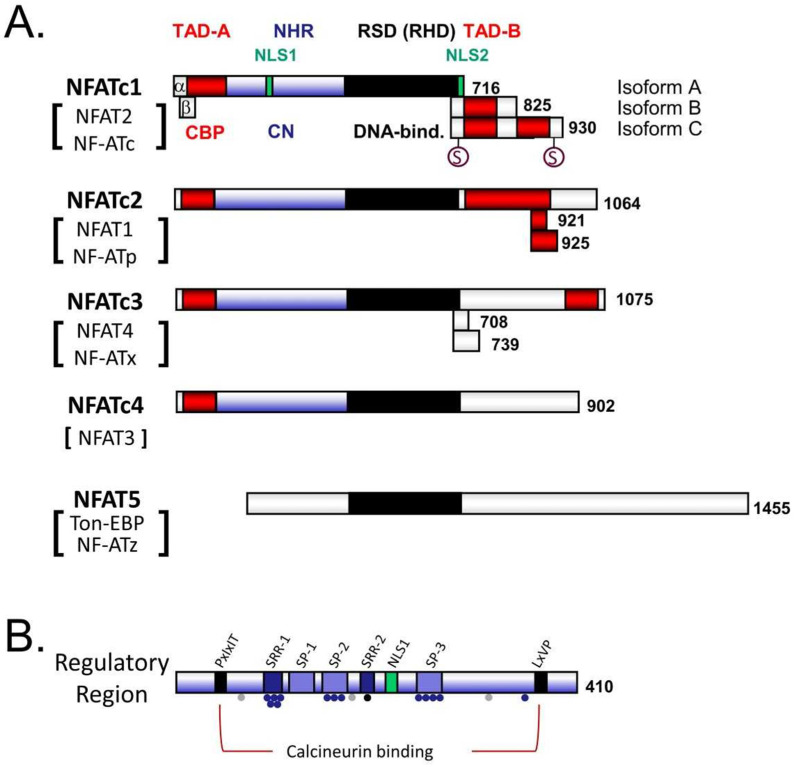
Schematic structure of the NFAT transcription factors. (**A**) Scheme of the ‘genuine’ Ca^++^/CN-dependent factors NFATc1, c2, c3 and c4, and of NFAT5. The DNA binding ‘Rel Similarity Domain’, RSD (or Rel Homology Domain, RHD), is shown in black, and the transactivation domains, TAD-A and TAD-B, are indicated in red. The NHR, ‘NFAT Homology Region’ or regulatory region, harbors the majority of phosphorylation motifs, including the ‘Nuclear Localisation Sequence’ 1, NLS1, and the two CN binding sites of consensus sequences PxIxIT and LxVP. S indicate two SUMOylation motifs in NFATc1 that are highly conserved in the other NFATs [21]. α and β in NFATc1 correspond to alternative N-terminal peptides that are generated by the transcription of exon1 (α, directed by the *Nfatc1* promoter P1) or exon 2 (β, directed by promoter 2). (**B**) Scheme of regulatory region of NFATc2 (modified according to ref. [24]). The serine-rich sequences SRR-1 and SRR-2, and serine-proline motifs SP-1, SP-2 and SP-3 are conserved among other NFATs. PxIxIT and LxVP are CN docking motifs. The filled circles indicate the phosphorylation sites. Upon stimulation, the majority of these site are de-phosphorylated (see ref. [24] for a detailed discussion).

The molecular cloning of the first NFAT factor, designated as NF-ATp, was described in 1993 by Anjana Rao’s laboratory (see ref. [14] for a personal account). Isolated from a murine T cell line by ammonium sulfate precipitation followed by heprin-agarose and oligonucleotide affinity chromatography, a protein of approximately 120 kD was identified that bound, in association with the AP-1 proteins Jun and Fos, to the distal purine box (the distal NFAT site) of the murine *Il2* promoter [15]. A similar, but distinct NFAT factor, designated as NF-ATc, was isolated in Jerry Crabtree’s laboratory from human Jurkat T cells [16]. Over a stretch of approximately 300 amino acid residues, this factor showed more than 70% sequence similarity to NF-ATp and—as the cloning of two further NFAT factors, designated NF-AT3 and NF-AT4 (or NF-ATx) [17,18], revealed—to the other two genuine members of the NFAT factor family. Although this domain shares less than 20% sequence similarity with the Rel DNA binding domain of NF-κB factors, its function as a DNA binding domain and organization in two loops and 10 β strands at positions similar to the Rel domain [19] led to its designation as a Rel Similarity (or Rel Homology) Domain (RSD or RHD). The RSD mediates the binding of NFATs to their canonical ‘core’ binding DNA motif A/T GGAAA. Contrary to the RSD, the other parts of NFATs are less well conserved among the four factors. However, short conserved motifs for the interaction with CN, with the co-factor CBP/p300, for nuclear import and export, phosphorylation and sumoylation have been identified in stretches near the 5′ and 3′ ends of both NFATc1, NFATc2 and the other NFATc proteins [20,21,22,23]. Those sequences located outside of RSD domains exhibited a strong transactivation activity when tested in co-transfection studies [20] (Figure 2).

**Note on the NFAT nomenclature:**

In November 2000, Dr. Ruth Lovering, Nomenclature Editor of the HUGO Gene Nomenclature Committee, sent “To all interested parties” a note to resolve the nomenclature of the NFATs. Following her proposal, the “Interested parties” should decide whether they prefer the nomenclature NFAT1, 2, 3, etc., or NFATc1, 2, 3. At the end of the vote, 19 “parties” supported the former and 20 the latter version (probably because these names were already in the HUGO database). According to this result, the HUGO Gene Nomenclature Committee proposed to use the designations NFATc1 (for NF-ATc or NFAT2), NFATc2 (for NF-ATp or NFAT1), NFATc3 (for NF-AT4 or NFATx), NFATc4 (for NF-AT3), and NFAT5 (for TonEBP, nfatz or NFATL1, an NFAT-related, Ca^++^-independent factor [25]). Unfortunately, the elder NFAT designations (NFAT1, etc.) are still used by many researchers in current publications. It would be a great help for the scientific community (in particular, for all who do not belong to the “Interested parties”) to follow the recommendations of the Nomenclature Committee.

## 2. NFAT Expression in Lymphocytes

All four genes of ‘genuine’ NFATc factors are transcribed in multiple RNA transcripts in numerous tissues from mice and humans [26]. In **conventional T lymphocytes**, only the three NFAT proteins NFATc1/NFAT2, NFATc2/NFAT1 and NFATc3/NFAT4 are expressed. In Western blots using specific Abs for the detection of NFATs in preparations of nuclear proteins from conventional peripheral T cells stimulated through their T cell receptor (TCR), NFATc1 and NFATc2 appear as the most prominent NFATs. At the protein level, NFATc2 is synthesized as one (or two) major protein(s), whereas NFATc1 proteins are more heterogeneous, forming mostly three prominent bands. Among those, one corresponds to NFATc1/αA, the short NFATc1 isoform that is strongly induced by TCR and further signals [27].

All three NFAT factors bind to very similar DNA motifs within the promoters and enhancers of their target genes that are trans-activated in DNA transfection assays. However, inactivation of individual *Nfatc* genes in mice resulted in quite diverging phenotypes. Whereas inactivation of the *Nfatc1* gene led to an early death of mice embryos in utero [28,29], *Nfatc2*-deficient mice were born at normal Mendelian ratios but developed with age a hyper-proliferative syndrome (i.e., a strong increase in lymphocyte proliferation) and elevated immune responses [30,31,32]. These earlier observations suggested that individual NFAT factors differ remarkably in their function to immune control reactions.

Immune receptor stimulation of conventional peripheral lymphocytes leads to rapid CN activation and de-phosphorylation of pre-formed cytosolic NFAT proteins. Within minutes, they are translocated into the nuclei of peripheral T and B cells and affect—together with further TFs—the expression of numerous genes. However, in addition to the induction of nuclear translocation, the transcription of the *Nfatc1* gene also becomes stimulated.

**Regulatory CD4^+^T cells,** Tregs, are generated both in thymus as so-called natural nTregs and in periphery from naive T cells as inducible iTregs. Due to their suppressive activity, they maintain immune homeostasis and prevent autoimmune diseases [33,34]. One mechanism through which Tregs suppress the activity of conventional T cells seems to be the gap junction-mediated transfer of cyclic adenosine monophosphate, cAMP, that inhibits T cell proliferation and occurs at high levels in nTregs [35]. High cAMP levels induce the accumulation of ICER/CREM, a transcriptional repressor that can bind to the *Il2* and *Nfatc1 P1* promoters, and suppress their induction in conventional CD4^+^T cells upon contact with nTregs [36].

In 2006, a report was published entitled “FOXP3 controls regulatory T cell function through cooperation with NFAT”. In this comprehensive study, an interaction between Foxp3, the ‘master regulator’ of Tregs [37], and NFATs was described, based on X-ray studies of DNA/protein complexes generated between the distal NFAT binding site of the *Il2* promoter, the RSD of NFATc2, and the forkhead domain of Foxp2 (a ‘sister factor’ of Foxp3) [38]. Based on those structural data and additional functional analyses, it was postulated—as mentioned in the headline of the publication—that the cooperation between Foxp3 and NFATs controls the activity of nTregs. However, in reports published before and after this study, we demonstrated that neither the simultaneous inactivation of NFATc2 and NFATc3 nor of NFATc1 and NFATc2 in murine T cells affected the inhibitory activity of nTregs to suppress conventional CD4^+^T cells in vivo and in vitro [39,40]. Instead, NFATs are necessary for the generation and stability of iTregs which differ markedly in their chromatin accessibility from nTregs [41]. In conventional CD4^+^T cells, the expression of NFATs—in particular, NFATc2 and NFATc3—is required for their suppression by nTregs [39], whereas in Tregs the induction of NFATc1/αA through TCR signals is inhibited by the binding of ICER and/or Foxp3 to the inducible *Nfatc1* P1 promoter [36,42].

A further mechanism of tolerance induction is the appearance of **anergic T cells** that are generated in response to partial or suboptimal T cell stimulation [43]. Anergic T cells remain alive for an extended period of time in a hypo-responsive state. One mechanism of generation of anergic T cells is sustained strong Ca^++^/CN signals that lead—without the contribution of co-stimulatory signals—to a cell-intrinsic program of self-inactivation [44,45]. Microarray analyses of genes that were induced in this way in D5 T and primary Th1 cells revealed an array of genes whose expression was strongly impaired in NFATc2-deficient mice. Their transcription is controlled by NFATc2 without the contribution of AP-1 (Jun/Fos) partner proteins [45]. In anergic T cells, sustained Ca^++^/CN signals can lead to the generation of NFATc2 homodimers that have been shown before to bind to NF-κB like binding motifs within the HIV and *Il8* promoters [46,47]. Such NF-κB-like motifs were also identified within the promoter of the anergy-inducing *Rnf128* (Grail) gene that, along with the *caspase 3* gene, contributes to the synthesis of E3 ubiquitin ligases Itch, Cbl-b and Grail and the ubiquitin-binding component of ESCRT complex. This network of expression of anergy-induced genes contributes to the functional unresponsiveness of T cells. Since the induction of NFATc1/αA depends on co-stimulatory signals, it is unlikely that the short NFATc1/αA isoform supports anergy induction. However, the longer P2-directed NFATc1 isoforms seem to control anergy (in B cells). Inactivation of NFATc1 in B cells that (over-)express the human oncogene TCL1 [48] led to a loss of anergy-inducing proteins, such as Cbl-b and Grail, and the generation of aggressive B-CLL cells [49]. Activation of murine splenic B cells for 24–48 h by anti-IgM antibodies resulted in a strong induction of NFATc1/αA, the short isoform of NFATc1 (see below) at the RNA and protein levels. By contrast, activation by LPS or anti-CD40—both inducers of NF-κB factors [50]—did not enhance but slightly suppressed NFATc1/αA transcription, whereas the double-treatment of B cells by anti-IgM and anti-CD40 enhanced NFATc1/αA expression [51,52]. Expression of human NFATc1/αA in chicken B cells suppressed their apoptosis [53], while NFATc2 supported activation-induced cell death, AICD, by stimulating the expression of Fas Ligand and/or other cell death-inducing molecules [54,55]. These data suggest that both NFAT factors play an important role in the fate of B lymphocytes.

## 3. Transcription of the Nfatc1 Gene

**Figure 3:**
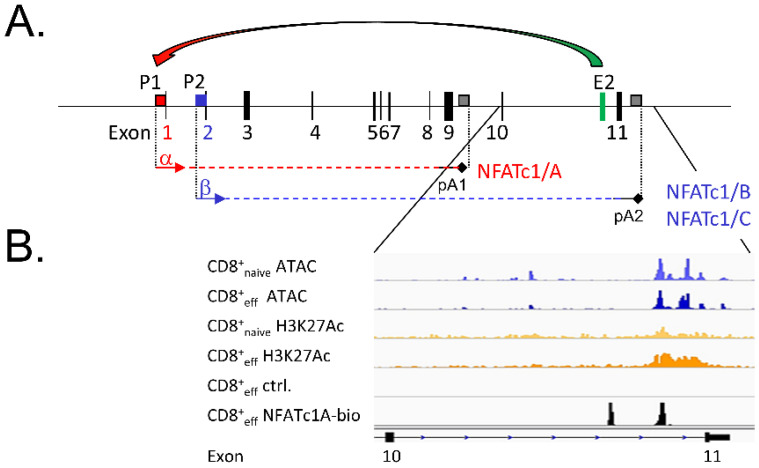
Organization of the murine *Nfatc1* gene and its remote intronic enhancer E2. (**A**) Scheme of the *Nfatc1* gene that harbors 11 exons, the inducible promoter P1 (located in front of exon1 [in red]), the constitutively active promoter P2 (in front of exon 2 [in blue]), two polyA addition sites, pA1 and pA2 (gray), and the remote E2 enhancer (green) in intron 10. In early DN thymocytes and resting lymphocytes, *Nfatc1* transcription is directed by promoter P2 resulting in transcripts that are polyadenylated at the polyA addition site pA2 (in blue). Upon activation, the start of *Nfatc1* transcription switches to P1 that directs short transcripts (polyadenylated at pA1) for the generation of isoform NFATc1/αA (in red). The P1 activity is strongly supported by the E2 enhancer activity (modified according to ref. [56]). Our analyses showed that the P2-directed long NFATc1 isoform NFATc1/C supports apoptosis, and NFATc1/αA supports the survival of T cells [57]. (**B**) Localization of the E2 enhancer within intron 10 of the *Nfatc1* gene. Open chromatin sites, as determined in ATAC assays [58], are shown, as well as H3K27Ac histone modifications [58], a molecular sign of active transcriptional enhancers [59], in naive and effector CD8^+^T cells. In the last line, results of Chip seq assays show the binding of NFATc1/A-bio protein to intron 10 DNA in CD8^+^ effector T cells [60]. The interplay of E2 activity with the P1 promoter is indicated by an arrow.

The **murine and human** ***Nfatc1*** **genes** consist of 11 exons and span approximately 110 and 134 kb DNA, respectively. In resting T cells—as in the majority of T cells from human blood—and in double-negative (DN) thymocytes, the *Nfatc1* gene is expressed in three prominent isoforms, the NFATc1/A, c1/B and c1/C proteins. Their expression is directed by the promoter 2 located in front of exon 2. Activation of T cells by TCR and co-stimulatory signals and, in DN thymocytes, by pre-TCR signals, leads to a rapid switch from the usage of promoter P2 to promoter P1 that is located in front of exon 1, and the proximal polyA site pA1 (Figure 3A).

Both *Nfatc1* promoters, P1 and P2, harbor DNase I hypersensitive chromatin sites and are characterized by CpG islands typical for the promoters of eukaryotic genes [61]. In lymphocytes, the CpG island of the P1 promoter is hypo-methylated, whereas it is hyper-methylated in non-lymphoid cells. In vitro methylation of P1 promoter DNA led to a marked decrease in its activity upon transfection into EL-4 T cells, suggesting an important role of CpG islands in *Nfatc1* gene expression [57]. The P1 promoter can be subdivided into two sequence blocks of approximately 270 bp each that show more than 80% sequence homology between mice and humans. Within the proximal block of homology there are two TATA-box like motifs that direct two transcriptional starts of the gene. There are numerous TF binding sites within both blocks of homology, including CREB/Fos/ATF2 and NFAT sites. No NFAT sites are situated within the P2 promoter. However, instead of these promoter/enhancer-like sequence features, the P1 promoter spanning approximately 800 bp of proximal DNA did not behave as the endogenous gene in transient transfections studies when linked to a test gene. In contrast to the *Il2* gene—but similar to the promoters of “Th2 genes” *Il4* and *Il5*—*Nfatc1* P1 promoter constructs (spanning between 0.8 and 5 kb of upstream DNA) were poorly induced in EL-4 T cells by TPA phorbolester and ionomycin treatment that mimic TCR signals. Instead, ionomycin and forskolin treatment exerted the strongest stimulatory effect on P1 induction. This implies that not all sequence elements for the induction of endogenous *Nfatc1* genes are located within promoter P1 [57].

For the identification of further sequence elements that control the induction of the *Nfatc1* gene, we investigated further DNase I hypersensitive sites (published by www.roadmapepigenomics.org, accessed on 17 March 2026). In addition to the promoter sites, we focused on two sites within exon 10 that we designated as E1 and E2. One of them, E2, appeared to be DNase I-sensitive only in CD3+ T cells. When we cloned approximately 1 kb of E2 DNA downstream in P1 (or P2)-driven luciferase test genes, we observed a strong increase in P1 (and mild in P2)-mediated luciferase activity upon transfection into EL-4 T cells by PMA and ionomycin induction [56]. No increase in P1 induction was detected by E1 constructs. Multiple NFAT binding sites are located within the E2 DNA segment, and mutation of these sites led to a strong decrease in enhancer activity, while mutation of other TF sites did not affect its activity [56]. These in vitro findings were corroborated in vivo by the deletion of E2 in transgenic mice bearing *Nfatc1-eGfp-Bac* constructs [52], and more recently by the analysis of *Nfatc1* transcription in T cells from *Nfatc1-E2^flx/flx^xCd4-cre* mice. In both mouse models the deletion of E2 enhancer led to a strong decrease in P1-driven NFATc1/A protein expression, whereas the expression of P2-driven NFATc1 proteins remained unaffected ([56] and unpublished data). These data demonstrate that the remote E2 enhancer strongly supports the induction of the *Nfatc1* P1 promoter by transmitting PI3 kinase-mediated signals [62] that mimic the induction of endogenous *Nfatc1* genes.

Under optimal stimulation conditions, the use of the P1 promoter leads to a splice from exon 1 to exon 3 and the use of the early polyA addition site, pA1, behind exon 9, in peripheral T and B cells [51,57] (Figure 3). We showed earlier that pA1 corresponds to a poor binding site for the cleavage stimulation protein CstF-64 which binds 5-fold stronger to transcripts from the distal polyA site pA2 as to those from A1 [63]. Since T cell activation results in a marked increase in splice/polyadenylation factors [64], one may conclude that in naïve or resting T cells the poor pA1 motif remains silent, while in activated T cells it is recognized, resulting in the generation of NFATc1/A transcripts [63].

The concentrations of TFs in the nuclei of eukaryotic cells are an important parameter of eukaryotic gene control. High levels of TFs lead to binding to weak, non-canonical sites of TFs and, thereby, to the activation of larger sets of target genes than low TF levels. This is also the case for genes, like the *Il2* gene that is predominantly or exclusively expressed in T lymphocytes. We will discuss below that the proximal *Il2* promoter/enhancer harbors multiple non-canonical sites for TFs that—as shown by their conversion to canonical sites—contribute to the T cell-specific expression of the *Il2* gene [13]. Similar non-canonical factor binding sites are a typical property of eukaryotic enhancers and allow the fine-tuning of eukaryotic gene expression (see ref. [65] for a recent discussion).

## 4. NFAT-Mediated Control of Lymphokine Gene Expression in Lymphocytes

The promoter/enhancer of murine and human *Il2* genes that span approximately 300 bp of the immediate upstream region [1,2,66] harbor two “strong” and several “weak” NFAT bindings sites [67,68]. The distal NFAT site around the position –285 corresponds to the ‘prototypical’ NFAT site (Figure 1) that has been used in numerous transfection and DNA–protein binding studies. NFAT binding was also observed to numerous other promoters/enhancers that control the induction of *Il3*+*Csf2* (GM-CSF), *Il4*, *Il5*, *Il13*, *Il17*, *Il21*, *Tnf* and *Ifn*γ genes upon activation in T cells [69,70,71,72,73,74,75,76,77,78,79,80,81]. While the binding of NFATs to these genes suggests that they might coordinately be expressed at certain stages of development, it does not mean that they are commonly transcribed in all types of T cells in vivo. Active transcription in vivo is not only orchestrated by the binding of single TFs but also by their (extracellular) activation and interplay with other factors, by the state of chromatin, the nuclear organization of the gene of interest, and further parameters.

One well-studied example of the interplay of NFATs with other TFs for faithful transcription in activated T cells is the ***Il2*****promoter/enhancer**. In vivo footprinting studies using resting and (TCR-) stimulated EL-4 T lymphoma cells (as non- and strong IL-producers) and 32 D premast cells (as poor producers) for the binding of TFs to the *Il2* promoter revealed the occupancy of “all” TFs—i.e., of NFATs, NF-κB, AP-1 and Octamefactors—only in activated T cells [82]. In resting EL-4 T cells, no factor binding was detected whereas in premast cells, only a part of the TFs was bound. In this scenario, the need for all TFs for full *Il2* induction in T cells might explain why this gene has been described as an ‘all or nothing’ gene that requires optimal threshold levels of TFs for its transcriptional induction [83]. Moreover, the T cell-specificity of *Il2* enhancer activity is controlled by the fine-tuned, coordinated binding of TFs. Several TF binding sites of enhancers belong to non-canonical sites and do not show a strong factor binding in DNA binding assays. Conversion of these non-canonical sites to consensus sites rendered the T cell-specific enhancer to an enhancer that was also active in non-T cells, such as in human HeLa cells and in Xenopus oocytes [13]. The analysis of the upstream region in the murine *Il2* gene in vivo, spanning the nucleotides up to position −8.4 kb, suggested that in addition to the proximal promoter/enhancer, additional control sequences orchestrate its T cell-specific expression in vivo [84]. They seem to contribute to the ‘poised’ state of the *Il2* locus in non-stimulated T cells that is marked by H3/K4 dimethylation of histones [85].

## 5. NFATs Control the Fate of B Lymphocytes

We also studied the role in apoptosis induction of NFATc1 isoforms by (over-)expressing either NFATc1/αA or NFATc1/βC in murine WEHI B lymphoma and chicken DT40 lymphoma cells. In both cell types, NFATc1/αA suppressed the B cell receptor-mediated apoptosis whereas NFATc1/βC slightly enhanced apoptosis [53]. Similar effects of both NFATc1 isoforms on apoptosis were observed earlier upon infection of primary T cells, whereas—like NFATc1/ßC—NFATc2 enhanced the apoptosis of T cells [57]. Although in T cell protein extracts NFATc2 (and NFATc1) was found to bind to the *Fasl* gene in Chip seq binding assays [60], the major effect of NFATc2 in Fas ligand induction seems to be executed through the induction of Egr2 and Egr3 factors and their binding to the *Fasl* promoter [86,87,88].

Transcriptome assays of B cells revealed a divergent effect of both ectopically expressed NFATc1 isoforms on numerous genes, such as on the *Aicda* and *Prdm1* genes encoding AID and Blimp-1, respectively, that control later steps of B cell differentiation in antagonistic ways. While NFATc1/αA enhanced the synthesis of *Aicda* RNA, it suppressed the synthesis of *Prdm1* RNA in WEHI cells. NFATc1/βC, on the other hand, suppressed *Aicda* RNA and enhanced *Prdm1* RNA synthesis. These observations indicate that individual NFATc1 isoforms differ markedly in the control of lymphocytes [53]. Novel findings on the predominant appearance of NFATc1/αA and its cooperative binding with IRF4 and BATF to almost 3000 genes in exhausted CD8^+^T cells in mice chronically infected with LCMV virus [89] suggest a novel, unexpected function for NFATc1/αA.

The ***Il4, Il5*****and** ***Il13*****genes** are predominantly expressed in Th2 cells. They are located within a stretch of 150 kb DNA in the genomes of mouse and man. While the *Il4* and *Il13 *genes are closely linked within 12 kb DNA and coordinately expressed in T and mast cells, the *Il5 *gene is located approximately 100 kb apart, separated by the *Rad50 *gene (that controls DNA repair). Generation of BAC transgenic mice bearing long segments of Th2 locus linked to an *Il4 *promoter/luciferase reporter gene led to the conclusion that a so-called ‘Locus Control Region’, LCR, is part of the *Rad50 *gene and orchestrates the proper expression of *Il4 *and *Il13 *genes in vivo, but not of the remote *Il5 *gene [90]. In CD4+T cells and natural killer cells, the LCR directs the promoters of Th2 cytokines to spatial proximity in a GATA3- and STAT6-dependent way [91], and deletion of one DNase I hypersensitive site of LCR led to a strong decrease in Th2 gene expression [92]. The binding to multiple sites within the promoters and enhancers of individual Th2-type genes, often in proximity to GATA3 sites [93], suggest that in collaboration with GATA-3 (and STAT6), NFATs contribute to the cell-type expression of *Il4, Il5 *and *Il13 *genes in Th2 cells. Inactivation of *Nfatc2 *and *Nfatc3 *genes in mice led to a strong increase in Th2-type lymphokine expression in naïve T cells suggesting an important (suppressive) role of both NFAT factors in Th2 cell differentiation [94].

NFATs control also the inducible tissue-specific expression of IL-3/GM-CSF locus in T cells and mast cells [95]. The ***Il3*****and** ***Csf2*****(GM-CSF) genes** are located 10.5 kb apart in a compact locus (and approximately 465 kb DNA upstream from the *Il5 *gene) and separated by an insulator element that is bound by CTCF and cohesion proteins [96]. While both genes are induced by similar signals in T cells and mast cells, they are individually expressed in other cells, such as in granulocytes and macrophages. However, in activated T cells the expression of both genes is strongly affected by NFATs [69,70,97].

## 6. NFAT Partner Proteins in T Cells

NFAT factors bind mostly in cooperation with other factors to DNA and, thereby, transactivate (or suppress) the transcription of their target genes. Although NFATs can also bind ‘alone’ as homodimers to DNA [46,47], the association with other factors in DNA binding is rather the rule than the exception [98]. Numerous genes that are activated in T cells through the Ca^++^/CN network harbor composite NFAT/AP-1 binding sites within their promoters/enhancers [99] that are bound by ternary NFAT/AP-1 complexes [100].

**AP-1 transcription factors** constitute a subgroup of a large family of basic region-leucine zipper (bZIP) factors that bind via a highly conserved stretch of basic amino acid residues to the TRE (TPA-responsive element) motif TGA C/T TCA in the promoters/enhancers of numerous inducible genes [19,101]. Their leucine zipper domain mediates the formation of homodimers and heterodimers with other zipper proteins [102], such as ATF and BATF factors [103,104]. In ternary AP-1 and NFAT complexes that consist of Jun/Fos dimers and NFATc2 monomers, the binding of NFATc2 to DNA is enhanced up to 10 fold [105]. The interaction between NFATs and Jun/Fos is predominantly mediated through contacts between the N-terminal half of NFAT’s RSD DNA binding domain and several amino acid residues within the zipper domains of c-Jun and c-Fos. Introduction of two mutations into the RSD of NFATc2 (R468A and T535G) abolished any cooperative binding and transacting activity of mutated NFATc2 with AP-1. Interestingly, only the induction of a set of NFAT target genes, *Il2*, *Il3*, *Csf2* (GM-CSF), *Il4*, *Ccl3* and *Fasl* genes, was inhibited whereas the induction of *Tnf* and *Il13* genes remained unaffected [106].

The group of AP-1 factors consist of three Jun (c-Jun, JunB and JunD) and four Fos proteins (c-Fos, FosB, Fra1 and Fra2). These proteins form homodimers (only between the Jun proteins) and heterodimers with other group members and further bZIP factors. While the interaction between the RSD domain of NFATs and the leucine zipper domains of c-Jun and c-Fos and their binding to composite NFAT/TRE motifs has been elucidated at the molecular level in great detail [19,107], the physiological role of complex formation between NFATs and various AP-1 factors during T (and B) cell differentiation is relatively poorly understood. Inhibition of interactions between NFATc2 and c-Jun/c-Fos led to a loss of NFAT binding to numerous target genes and the induction of “a limited set of anergy-associated genes” [45]. Among those are genes encoding E3 ubiquitin ligases and proteases which affect the proper signaling upon TCR stimulation [108]. Isolation of partner proteins binding to biotin-tagged NFAT proteins in (non-stimulated) human Jurkat T cells revealed the binding of JUNB, CREB1 and ATF7 to both (full-length) NFATc1 and NFATc2, whereas c-JUN, c-FOS and RUNX1 bound only to NFATc2 [109]. In a similar approach using murine WEHI 231 B cells, the interferon responsive factors IRF4 and IRF7 were identified as NFATc1/A binding partners [110].

In former approaches, the p21^SNFT^protein (21 kDa small nuclear factors isolated from T cells) was identified as a further NFAT partner protein of the bZIP factor family [111]. p21^SNFT^(designated today as BATF3) is a member of **BATF factors** that, due to the lack of a transacting domain, can act as repressors of AP-1. By its binding to NFATc2, BATF3 can block the induction of *Il2* promoter in T cells. In addition to binding to Jun proteins, BATF factors can also bind via their leucine zipper domain to IRF4 and, therefore, positively affect transcription [112,113].

**IRF4** was described previously to bind to NFATc2 and, thereby, to control the activity of the *Il4* promoter in T cells [114]. In a more recent report, IRF4 has been described as a factor that supports—along with NFATc1 and BATF—the exhaustion of CD8^+^T cells during tumor formation [89]. However, overexpressing IRF4 and BATF suggested that both factors cooperate and counteract the exhaustion of tumor-infiltrating CAR (chimeric antigen receptor) T cells, leading to better tumor control. These contradictory findings suggest that, like other factors (the NFATs), the mode of complex formation and the cellular context decides whether BATF and IRF4 complexes (along with their complex formation with NFATs) act as transcriptional activators or suppressors [115].

There are numerous reports on further NFAT partner proteins in both lymphocytes and non-lymphoid cells (see ref. [98] for a compilation). As NFAT partners in lymphocytes, c-Maf and NIP45 were described to contribute to *Il4* [54,116], and CBP/p300, ICER, EGR1 and EGR4 to *Il2* and *Tnf* transcription [20,117,118,119]. It is worth noting that apart from the interaction of GATA-3 with NFATs in the control of IL-5 transcription [120], no intimate direct interactions have been detected between other ‘master regulators’ of T cell development, such as Tbx21 and Rorγt, with NFATs. However, NFATs orchestrate many steps of T cell development, either by the transcriptional control of genes encoding ‘master regulators’, such as the *Ror*γ*t* gene [121], or by the expression of many cytokines that control T cell development (see above and the discussion in refs. [122,123]).

## 7. Calcineurin (CN) and Canonical NFAT Activation

The identification of NFATs as CsA targets [3,4] leads to the question as to through which mechanisms CsA inhibits the activity of NFAT factors in T cells. The isolation of a newly cloned immunophilin, designated as cypC [124], and its use in affinity chromatography approaches with protein extracts from T cells treated with CsA, resulted in the identification of a 55 kD protein and two smaller proteins as CsA targets. While the 55 kD protein corresponds to the large catalytic subunit of CN, CNA, the smaller proteins correspond to CNB, the regulatory subunit of CN, and calmodulin [10,124]. Soon after, it was shown that CNA supports NFATs’ nuclear translocation and the activity of the *Il2* promoter by NFATs [11,12]. These findings suggested that the NFATs are direct targets of Ca^++^-dependent protein phosphatase CN [125].

CN is a hetero-trimer in vivo, consisting of the large catalytic subunit A (either as CNAα, CNAβ or CNAγ), the smaller regulatory subunit B (CNb1 or CNb2), and calmodulin. In two experimental systems, inactivation of genes encoding either CNAα or CNAß led to mild effects in peripheral lymphocytes, likely due to compensation by the other subunit [126,127]. In a further approach, impaired proliferation and cytokine production was reported for peripheral T cells from CNAα-deficient mice [128]. By contrast, inactivation of the *Ppp3r1* gene encoding Cnb1 resulted in strong defects in positive thymocyte selection and intestinal immune homeostasis [129,130]. CNA binds to two sites within the N-terminal half of NFATs. One, the CN binding site A, harbors the sequence SPRIEITPS at amino acid position 110–118 (within NFATc2) that includes the core docking motif PxIxIT [131,132]. The CN binding site B spans the core motif LxVP near the 3’ end of the N-terminal half of NFATs [22]. In recent comprehensive screens to detect uncovered CN interactions in human cells, both binding sites of NFATs were also identified as CN docking sites and, thereby, confirmed the earlier findings [133].

In naïve T lymphocytes, NFATs are localized within the cytosol as highly phosphorylated proteins in large RNA-protein scaffold complexes containing NRON, a non-coding RNA repressor, the leucine-rich repeat kinase LRRK2, cytoplasmic scaffold proteins of the Homer family, and further proteins [134,135,136,137]. For NFATc2, at least 21 phosphoserine residues have been mapped, 18 of them within the N-terminal regulatory domain spanning the amino acid residues 99–398 [24]. A total of 14 out of 18 phosphoserine residues are localized within five conserved phosphorylation motifs that, in addition to the CNA docking motif SPRIEIT around amino acid position 110 and the nuclear localization signal (NLS) KRR at position 253–255, are part of the regulatory domain (Figure 2B). Introduction of alanine mutations in all conserved serine motifs followed by functional tests indicated five (out of seven) serine residues within the SRR1 motif as the most important (de-) phosphorylation motifs that orchestrate—upon de-phosphorylation—the exposure of the NLS motif for NFAT nuclear transport [24]. The SRR1 motif was identified as a target of casein kinase 1 (CK1) that is bound to NFATs in resting T cells and dissociated upon activation [138]. However, the de-phosphorylation of at least 13 of 14 conserved serine residues and the phosphorylation of SSPS peptide at position 53–56 near the N-terminal end (in the N-terminal “β” peptide, see Figure 2B) appeared to be necessary for the nuclear translocation and full transcriptional activation of NFATc2 [24].

The ‘canonical’ induction of NFAT by Ca^++^/CN signals certainly represents one of the pillars of activation of lymphocytes by antigen-driven signals (see refs. [23,125] as reviews). In T cells, triggering of the TCR complex leads to the rapid induction of several typrosine protein kinases that rapidly results in the generation of two second messengers, inositol-1,4,5-triphosphate (IP_3_) and diacylglycerol (DAG). IP_3_ binds to and opens receptors at the endoplasmic reticulum (ER), which is followed by the release of Ca^++^ from the ER. The Ca^++^ release triggers the activation of stromal interacting molecules STIM1 and STIM2 that contact the ORAI proteins ORAI1 and ORAI2 at the cell membrane.

Both ORAIs are components of Ca^++^ release-activated channels (CRACs) that, upon activation, allow the entry of extracellular Ca^++^. This process, called SOCE (Store Operated Ca^++^ Entry), results in a marked increase in intracellular Ca^++^ that binds to calmodulin (and further Ca^++^ binding proteins) and activates CN, followed by the de-phosphorylation of cytosolic NFATs and their nuclear translocation (see refs. [139,140] as reviews).

## 8. Alternative Ways of NFAT Activation

In addition to the Ca^++^/CN-mediated NFAT activation, there are other signals that activate NFATs in lymphocytes. When Amiya Patra studied in our laboratory the induction of NFATs in murine thymocytes, he observed a gradual increase in nuclear NFATc1 proteins in early thymocytes from the double negative DN1 to DN3 thymocyte stages [141]. These NFATc1 proteins were generated from P2 promoter-derived *Nfatc1* transcripts that were synthesized in early DN thymocytes. This increase in NFATc1 levels in DN thymocytes is stimulated by IL-7 and resistant to CsA. In vitro phosphorylation assays suggested a direct phosphorylation of NFATc1 at amino acid position Tyr374 by Jak3 [141]. The generation of pre-TCR in late DN3 stages correlates with the formation of NFATc1/A transcripts that, due to the induction of the remote enhancer E2, are generated by the switch from P2 to P1 promoter activity [56].

There are reports that IL-2 treatment of naive peripheral CD8^+^ T cells in vitro for 3 d and longer induces NFAT activation in a CsA-independent way [142] (which we could confirm; E.S., unpublished data). However, double-treatment of Stim-deficient splenic T cells by IL-2 and IL-7 rescued their proliferation to be more efficient than IL-2 alone, with the expression of numerous genes associated with aerobic glycolysis, such as the *Slc2a1*, *Slc2a3*, *Hk2*, *Myc* and *Irf4* genes, and the nuclear translocation of NFATs [143]. Since in Stim-deficient T cells IL-2 and IL-7 also rescued the phosphorylation of mTOR and of ribosomal S6 protein (a target of PI3K-AKT-mTOR signals), one may conclude that in lymphocytes—in addition to Ca^++^/CN signals—NFATs can also be activated through the PI3K-AKT-mTOR pathway [143]. This is supported by the findings that IL-2- and IL-7-mediated signals can act on CD8^+^T cells to upregulate the expression of GLUT1, one of the glucose transporters, to increase the uptake of glucose for aerobic glycolysis [144,145].

## 9. NFATs and the Metabolism of T Cells

The activation of naïve T lymphocytes to effector T cells is closely linked to changes in their cellular metabolism [146,147]. The transcriptional induction of NFATc1 and its function depends on the ‘metabolic reprogramming’ of naïve cells, whereas NFATs control the expression of numerous genes that orchestrate metabolic changes during T cell activation. Thus, a complex network exists between NFAT activity and the cellular metabolism of lymphocytes that “should be considered as a driving force in protecting us from infection and cancer” [145].

In naïve lymphocytes, energy is mainly provided by oxidative phosphorylation (OXPHOS), whereas upon TCR-mediated activation, T cells switch their metabolism largely from OXPHOS to aerobic glycolysis. This provides lymphocytes both with ATP and building blocks for their rapid cellular growth and proliferation [148,149]. When we studied the expression of genes encoding transporters and enzymes that orchestrate aerobic glycolysis in NFATc1-deficient splenic CD8^+^T cells, upon activation we detected a marked decrease in RNA levels in almost all genes compared to wild type mice [60]. In particular, the expression of *Slc2a1* and *Slc2a3* genes encoding the glucose transporters GLUT1 and GLUT3, and the expression of the *Hk2* gene encoding hexokinase 2, decreased strongly in the absence of NFATc1—but not of NFATc2—in CD8^+^T cells. The *Slc2a3* and *Hk2* genes are direct targets of NFATc1, since, in Chip seq assays using CD8^+^T cells, the binding of NFATc1 (and NFATc2) was detected to both genes [60,150]. By contrast, the majority of genes encoding enzymes of the glycolytic pathway, including *Slc2a1*, did not show a robust binding of NFATc1. Therefore, the majority of genes orchestrating aerobic glycolysis are not direct NFAT targets but seem to be controlled indirectly by NFATc1. This was shown by the analyses of CD8^+^T cells from mice (over-)expressing a constitutively active (ca) version of NFATc1 [151] that enhanced not only *Slc2a3* and *Hk2* but also *Slc1a1*, *Irf4* and *Hif1a* expression, as well as rescuing the decrease in glucose uptake in SOCE-deficient T cells [143].

Similar results of NFATc1 activity on aerobic glycolysis were also described recently by Boztug and colleagues for human CD8^+^T cells from three patients suffering from recurrent infections, decreased antibody responses, etc. This suggests an important role of NFATc1 in human immune responses [152].

Previous studies demonstrated that in Th17 cells, the promoter of the *Il17a* gene is bound and induced by NFATc1 [153]. Interestingly, the ablation of GLUT3 exerted a strong negative effect on the effector function of Th17 cells in models of autoimmune colitis and encephalomyelitis, and revealed a link between GLUT3 expression and the generation of acetyl-CoA through ATP-citrate lyase (ACLY). These findings suggest that the generation of cytosolic acetyl-CoA (downstream of GLUT3) is a critical metabolic checkpoint for Th17 immune responses that affects histone acetylation [154,155].

The induction of aerobic glycolysis is only one step in the complex network of the ‘metabolic re-programming’ of naive T cells upon activation [156,157]. Both glycolysis and fatty acid oxidation (FAO) feed the tricarboxylic acid (TCA) cycle via acetyl-CoA whereas glutaminolysis, on the other hand, fuels the TCA cycle via α-ketoglutarate upon conversion of glutamine to glutamate [158]. The TCA cycle is closely associated with the electron transport chain (ETC) in mitochondria, the main ATP energy source for resting lymphocytes. Similar to 2-deoxygucose (2-DG), low concentrations of oligomycin, an inhibitor of ETC, strongly inhibited the induction of NFATc1/A (E.S., upublished), suggesting that the ATP supply by aerobic glycolysis and ETC is necessary for NFATc1/A induction.

The production of energy is accompanied with the generation of reactive oxygen species (ROS) which can drive T cells to apoptosis at high levels [159]. However, there is growing evidence that ROS can also act as intracellular signaling molecules, and NFATs are important targets of ROS in lymphocytes [156]. Low levels of ROS that are generated by mitochondrial OXPHOS and the catalytic activity of NADPH oxidase (NOX) during T cell activation enhance TCR signals and NFAT activation [160,161,162]. Further targets of ROS are c-Myc, mTOR and Hif-1α that are either NFAT targets and/or closely associated with NFAT activation [156,159,163,164].

## 10. NFATs as Oncoproteins and Tumor Suppressors

Transplant patients who were treated for years with CsA to prevent organ rejection showed a marked increase in tumor formation [165,166]. Since NFATs are primary targets of CsA inhibition, one may assume that the poor CD8^+^T cell immunosurveillance of neoplastic cells is mediated by the inhibition of NFAT activity.

The murine *Nfatc1* and *Nfatc3* genes were previously identified as common insertion sites (CISs) for oncogenic viruses [167], and all insertions were found either close to the promoter or enhancer regions [168], mostly leading to NFAT suppression. Since infections of newborn mice with the T cell lymphomagenic retrovirus SL3-3 MLV led to an acceleration of lymphoma generation in NFATc3-deficient mice, NFATc3 corresponds to a tumor suppressor directed against the generation of virus-induced lymphoid tumors [169]. Investigations of NFATc2-deficient mice which develop chondrocytic neoplasms led to a similar conclusion, i.e., that NFATc2 can act as a tumor suppressor for the generation of chondrocytic malignancies [170].

In vitro assays suggested previously that NFATs can exert dual properties in carcinogenesis. Transformation of pre-adipocytes by a constitutively active (ca) version of NFATc1/A showed that caNFATc1/A acts as an oncogene for the development of human adipocytic and other tumors [171]. When caNFATc and caNFATc2 were over-expressed in NIH 3T3 cells, caNFATc2 induced cell cycle arrest and apoptosis, whereas caNFATc1—corresponding to caNFATc1/αA—led to increased proliferation and cell transformation [172]. A similar functional dichotomy was observed for two constitutively active versions of NFATc1 that differed only in their N-termini: while caNFATc1/αA-expressing exon 1 (and not exon 2) induced the oncogenic transformation of NIH 3T3 cells, caNFATc1/βA-expressing exon 2 (instead of exon 1: see Figure 2A) reduced the proliferation and induced cell death [173].

Due to the ectopic (aberrant) expression of individual NFAT members in various types of cancer stem cells, NFATs seem to play important roles in the tumor formation of numerous types of non-lymphoid cells (see refs. [98,174,175] as compilations). NFATc2 has been described to enhance the invasion and growth of glioma cells [176,177], to control the invasion and migration of human lung cancer cells [178], and to promote the invasion and metastasis of colorectal cancer cells [179]. NFATc1 is overexpressed in pancreatic cancer cells, and its ectopic (over-)expression enhanced the expression of c-Myc and cancer progression [180]. The interplay of NFATc1 with further signaling ways, as with STAT3 and Sox2, promotes pancreatic cancer initiation and cancer cell plasticity [151,181].

## 11. NFATs and the Exhaustion of Cytotoxic CD8^+^T Cells

One mechanism that impairs the anti-tumor activity of the immune system is the generation of dysfunctional **‘exhausted’ CD8^+^T cells**. Such T_EX_ cells exhibit a reduced proliferative capacity, increased expression of co-inhibitory receptors and type I interferons, and resistance against checkpoint immune therapies [182,183]. In a comprehensive study on the role of NFATs in the generation of T_EX_ cells, Martinez et al. [150] infected CD8^+^T cells with retroviruses expressing a constitutive active version of NFATc2 (NFAT1) bearing three mutations within the RSD domain of NFATc2 that abolished any interaction with the co-factor AP-1. Upon expansion in vitro by IL-2, the transfected CD8^+^T cells were injected into mice followed by infection with bacteria expressing the gp33 epitope (that is recognized by the P14 receptor of transferred CD8^+^T cells). After 3–5 days, the injected cells were isolated and investigated. Compared to the control mice, the mice injected with CD8^+^T cells expressing mutated NFATc2 showed a poor virus recovery, and the injected CD8^+^T cells a strong increase in PD-1, LAG3 and TIM3 expression. A similar approach was used to study the effect of NFATc2 mutation on CD26 tumor formation expressing influenza hemagglutinin (HA) [184]. Again, on CD8^+^T cells infected with viruses expressing mutated NFATc2 and injected into tumor-bearing mice, a strong increase in inhibitory receptors PD-1, LAG3 and TIM3 was observed, and a defect in anti-tumor activity, as determined by the poor tumor rejection of mice injected with CD8^+^T cells expressing mutated NFATc2 [150]. These data suggest that the full activation of NFATc2 (and of further NFATs, such as NFATc1-AP-1) complexes support anti-tumor activity, whereas partial activation—without AP-1—contributes to the exhaustion of CD8^+^T cells [150].

However, other findings—including those of our own approaches—cast doubts on the generalization of these findings. In one of the first comprehensive studies on gene expression using DNA microcarrays for analyzing murine CD8^+^T cells exhausted upon chronic LCMV infection, the expression of the *Nfatc1* gene was markedly enhanced compared to its expression in effector, memory and naive T cells [185]. Moreover, numerous genes encoding inhibitory receptors, such as the *Pdcd1* (PD-1), *Ctla4* and *Lag3* genes, are known as NFATc1 targets [60,150,186,187], and the analysis of proteins from exhausted CD8^+^T cells generated in vitro or in vivo revealed the predominant expression of NFATc1/αA in the nuclei of exhausted murine CD8^+^ T_EX_ cells [89]. In the nuclei of T_EX_ cells generated in vitro for 6–8 days by persistent anti-CD3 stimulation or by co-incubation of OT-I CD8^+^T cells with B16 tumor cells, the predominant expression of NFATc1/αA and the marked decline in NFATc2 levels was observed. Moreover—as the induction of CD8^+^T cells from δE2 (*Nfatc1-E2 ^flx/flx^ x Cd4-cre*) mice suggest—the induction of NFATc1/αA suppresses *Nfatc2* under chronic stimulation conditions (Ref. [188] and unpublished data). Due to these findings, we speculate that the NFATc1/αA induction contributes to the survival of T_EX_ cells under the chronic stimulation conditions of cancer formation.

## 12. Outlook: NFATs as Molecular Targets for Tumor Therapies

The central role of NFATs in the activity of cytotoxic T cells suggests that NFATs play a decisive role **in the re-activation of TILs in checkpoint therapy approaches.** Cytotoxic CD8^+^T cells are key components of an adaptive immune system that kill both malignant and infected cells. Since to a large degree their activity is mediated by the Ca^++^/CN/NFAT network, studying NFAT activity in cytotoxic CD8^+^T cells during carcinogenesis and infections is of crucial importance to understand these diseases at the molecular level. Tumor cells and chronically infected cells persistently deliver signals to impair the activity of the immune system. The success of novel immune checkpoint therapies is based on the use of antibodies that interfere with those signals.

The intimate contacts between tumor cells and tumor-infiltrating lymphocytes (TILs) results in the competition between tumor cells and TILs for nutrition, such as for glucose [189]. It is likely—but not proven—that the competition between tumor cells and TILs for metabolites affects the induction of NFATs in TILs. Other mechanisms through which tumor cells could affect the activity of NFATs in TILs are the secretion of lactic acid and of ROS (Reactive Oxygen Species) molecules [159,190].

Numerous types of tumor cells secrete lactic acid into the culture medium [191], and studies on tumor patients revealed a positive correlation between tumor burden and lactic acid secretion into circulation [192]. Previous studies revealed a strong inhibitory effect of lactic acid on the proliferation, cytokine production and cytotoxic function of both human and murine cytotoxic CD8^+^T cells which seems to be mediated through the inhibition of NFATs. While in in vitro tests, lactic acid inhibited the activation of NFATs in T cells, the activation of NF-κB and of further signaling molecules remained unaffected [190]. This effect is mediated through the acidification of the tumor micro environment (TME): while equal concentrations of lactate did not affect cytokine production, the acidification of the medium by lactic acid (to a pH of 6.4 typical for 15 mM lactic acid) inhibited the production of IL-2 and Ifn-γ. These data indicate that the production of lactic acid by tumor cells blunts the anti-tumor activity of cytotoxic CD8^+^T cells and, thereby, contributes to the escape of tumor cells from immunosurveillance [190,192,193].

ROS are ‘side products’ of the mitochondrial electron transport chain (ETC) and of membrane-bound NADPH oxidase (NOX) [156]. Previous studies showed that ETC-generated ROS support NFAT activation and IL-2 production, albeit at relatively low concentrations [160], whereas high ROS concentrations that support tumor growth [194] interfere with T cell activation. One target of ROS is CN which is oxidized in its active center by ROS [195].

These examples illustrate how tumor cells could dampen the activity of CD8^+^ T cells through NFATs. It remains to be shown in detail whether and how NFAT factors that control the activity of CD8^+^ T cells are involved in this process. More studies are necessary to elucidate the molecular events that might be involved in the suppression of NFAT activity in CD8^+^T cells by tumor cells.

## 13. Conclusions

NFATc1 and NFATc2, the most prominent NFAT factors in the nuclei of activated T cells, share numerous properties. This led to the view that both transcription factors exert similar functions in lymphocytes. However, their synthesis and activity differ markedly in peripheral T cells. While persistent antigen-signals lead to ‘tonic’ synthesis of NFATc2, strong antigen signals result in a switch of synthesis of ‘long’ NFATc1 protein to the short isoform NFATc1/aA which lacks a conserved C-terminal peptide of approximately 250 amino acids. NFATc1/aA is the most prominent NFATc1 protein in exhausted CD8^+^ T cells that (i) dampens the expression of NFATc2 and (ii) supports the survival of exhausted cells. Thereby, NFATc1 plays an individual role in control of peripheral CD8^+^ T cells that favours this factor as a molecular target in tumour therapies.

## Data Availability

No new data were created or analyzed in this study. Data sharing is not applicable.
